# A mendelian randomization study investigates the causal relationship between immune cell phenotypes and cerebral aneurysm

**DOI:** 10.3389/fgene.2024.1333855

**Published:** 2024-01-19

**Authors:** Xingjie Shi, Tao Wang, Da Teng, Shiqiang Hou, Ning Lin

**Affiliations:** ^1^ Department of Neurosurgery, The Affiliated Chuzhou Hospital of Anhui Medical University, The First People’s Hospital of Chuzhou, Chuzhou, Anhui, China; ^2^ Department of Neurosurgery, Xishan People’s Hospital, Wuxi Branch of Zhongshan Hospital Affiliated to Southeast University, Wuxi, Jiangsu, China; ^3^ Department of General Surgery, The Affiliated Chuzhou Hospital of Anhui Medical University, The First People’s Hospital of Chuzhou, Chuzhou, Anhui, China

**Keywords:** cerebral aneurysms, immune cell phenotypes, MR analysis, causal association, NK cell

## Abstract

**Background:** Cerebral aneurysms (CAs) are a significant cerebrovascular ailment with a multifaceted etiology influenced by various factors including heredity and environment. This study aimed to explore the possible link between different types of immune cells and the occurrence of CAs.

**Methods:** We analyzed the connection between 731 immune cell signatures and the risk of CAs by using publicly available genetic data. The analysis included four immune features, specifically median brightness levels (MBL), proportionate cell (PC), definite cell (DC), and morphological attributes (MA). Mendelian randomization (MR) analysis was conducted using the instrumental variables (IVs) derived from the genetic variation linked to CAs.

**Results:** After multiple test adjustment based on the FDR method, the inverse variance weighted (IVW) method revealed that 3 immune cell phenotypes were linked to the risk of CAs. These included CD45 on HLA DR+NK (odds ratio (OR), 1.116; 95% confidence interval (CI), 1.001–1.244; *p* = 0.0489), CX3CR1 on CD14^−^ CD16^−^ (OR, 0.973; 95% CI, 0.948–0.999; *p* = 0.0447). An immune cell phenotype CD16^−^ CD56 on NK was found to have a significant association with the risk of CAs in reverse MR study (OR, 0.950; 95% CI, 0.911–0.990; *p* = 0.0156).

**Conclusion:** Our investigation has yielded findings that support a substantial genetic link between immune cells and CAs, thereby suggesting possible implications for future clinical interventions.

## Introduction

Localized dilations resembling balloons in major branches of brain arteries, known as CAs, are found in about 3.2% of the population (Vlak et al., 2011). Aneurysmal subarachnoid hemorrhage (aSAH), which is an extremely dangerous type of stroke, can occur when aneurysms rupture. Around one-third of aSAH patients face death, while another third achieve complete recovery, and the remaining third show significant dependency, suggesting an unfavorable outlook (Nieuwkamp et al., 2009). Despite a heritability estimate of 41% for aSAH derived from a prior twin study (Korja et al., 2010), the etiology of brain aneurysms remains inadequately comprehended.

Lately, there has been significant fascination with the connection between immune cells and various illnesses. Studies have determined a cause-and-effect relationship between the number of immune cells in the outer regions of the body and atrial fibrillation. Further analysis of specific groups has shown that increased levels of CD4^+^ T cells are associated with a higher likelihood of developing atrial fibrillation (Feng et al., 2022). Furthermore, NKT cells play a crucial part in the development of multiple sclerosis (He et al., 2022). A recent investigation revealed that endothelial cells play a crucial role in the susceptibility to CAs. The discovery was made by conducting a meta-analysis of genome-wide association studies (GWAS) across different European and East Asian countries, involving 10,754 cases and 306,882 controls of various racial backgrounds. Additionally, the study revealed that smoking and susceptibility to blood pressure were prominent genetic risk factors for CAs (Bakker et al., 2021). Moreover, mounting proof indicates that immune cells inside CAs have a vital function in their formation and eventual bursting. According to a study, there is evidence indicating an inequilibrium in Th17 and Treg cells among people with CAs. Additionally, there is a direct relationship between the frequency of Th17 cells and the seriousness of spontaneous subarachnoid hemorrhage (SAH) (Song et al., 2021). Furthermore, impaired regulatory T cell function has been observed in CAs (Zhang et al., 2018; Zhao et al., 2018). Nevertheless, the presence of a conclusive cause-and-effect connection between CAs and the characteristics of immune cells remains ambiguous.

The utilization of genetic variation as independent variables in MR is a methodology employed to examine the causal relationship between exposure and outcome. The possible correlations between genetic variation associated with exposure and outcome may suggest the influence of exposure on the outcome (Lawlor et al., 2008). Since genetic variations are allocated randomly during conception, this effect remains unaltered by confounding factors and reverse causation. As a result, we utilized recently released combined information on immune cells and combined data from a GWAS on midbrain aneurysms to explore the causal link between immune cell characteristics and CAs using a two-sample MR method in this study.

## Methods

### Sources of data for exposure and outcome

Single nucleotide polymorphisms (SNPs) linked to CAs were obtained from a vast GWAS dataset comprising 945 cases and 472,738 controls of European descent (Sakaue et al., 2021). Due to the lack of personal information in this data, we do not consider the impact of age, gender, etc. on the results. The GWAS Catalog provides publicly available summary statistics for each immune trait, with accession numbers ranging from GCST90001391 to GCST90002121 (Orrù et al., 2020). The dataset includes a total of 731 phenotypes of immune cells, which consist of 118 absolute cell counts, 389 median fluorescence intensities indicating surface antigen levels, 32 morphological parameters, and 192 relative cell counts. To ensure that there were no overlapping cohorts, the initial GWAS on immune traits utilized information from 3,757 individuals of European ancestry. A Sardinian sequence-based reference panel was used to impute genotypes for around 22 million SNPs, utilizing high-density arrays. Associations were then assessed after adjusting for covariates (Sidore et al., 2015; Wang et al., 2023).

### Selection of IVs

The IVs were screened using the following criteria: (Vlak et al., 2011): Choosing IVs with a significance level below: *p* < 1.0 × 10^−5^; (Nieuwkamp et al., 2009); Applying a clumping window size of 10,000 kb and a threshold of R^2 < 0.001 to reduce linkage imbalance (LD) and avoid bias in the outcomes. It is essential to attribute the impact of SNPs on outcome and exposure to only one allele during MR analysis. Consequently, SNPs exhibiting a palindromic structure were excluded. The F value, calculated as (R^2 (n-k-1))/(k (1-R^2)), is frequently used to evaluate the degree of correlation between the exposure and IVs. In this context, “n” stands for the quantity of exposure samples in the GWAS, “k” indicates the number of IVs, and “R^2” represents the degree to which IVs explain the variation in exposure. Typically, a F statistic lower than 10 is considered as a sign of a weak independent variable, which could potentially affect the results of the study. Then, 508 IVs for CAs were obtained for later analysis, Supplementary Tables S1–S22 contained details about the specific IVs. In addition, our research utilized reverse MR analysis to investigate the causal influence of CAs on phenotypes of immune cells.

### Statisitical analysis

To assess the causal connection between 731 immune cell phenotypes and CAs, several techniques were employed including IVW, MR-Egger, weighted median, weighted mode and simple mode. Furthermore, the Cochran’s Q statistic and its corresponding *p*-values were utilized to evaluate the heterogeneity among the chosen independent variables. In order to account for possible horizontal pleiotropy, MR-Egger was employed to assess whether the SNPs included in the study demonstrated such effects (Burgess and Thompson, 2017). However, when the MR-Egger method found significant outliers, we used the MR-PRESSO as the primary analysis because the regression accuracy of the MR-Egger was generally lower than that of the MR-PRESSO outlier test (Burgess and Thompson, 2017), and the MR-PRESSO method was utilized to address the possibility of pleiotropy by considering the MR pleiotropic residuals and outliers (Verbanck et al., 2018; He et al., 2022). We then applied the weighted median method, which can also estimate causality, but only if it comes from at least 50% of valid IVs (Bowden et al., 2016). Additionally, the accuracy of simple model is lower than that of weighted model, both of which use causal estimates from a single SNP to form clusters, but weighted model assigns weight to each SNP, but they may be superior to MR-Egger in detecting causal effects (Hartwig et al., 2017; Zuo and Li, 2023). To evaluate the correlation between specific SNPs and the obtained outcomes, a Leave-one-out (LOO) sensitivity analysis was utilized in the study (Burgess et al., 2017). This analysis aimed to determine if any single SNP was driving the association. Furthermore, scatter diagrams were employed to evaluate the influence of anomalies on the findings, whereas funnel diagrams were used to assess the reliability of the correlation and the lack of variability. The MR Analysis was conducted using the R (version 4.2.1) software package TwoSampleMR.

## Results

### Genetic prediction of immunophenotypes on CAs

**FIGURE 1:**
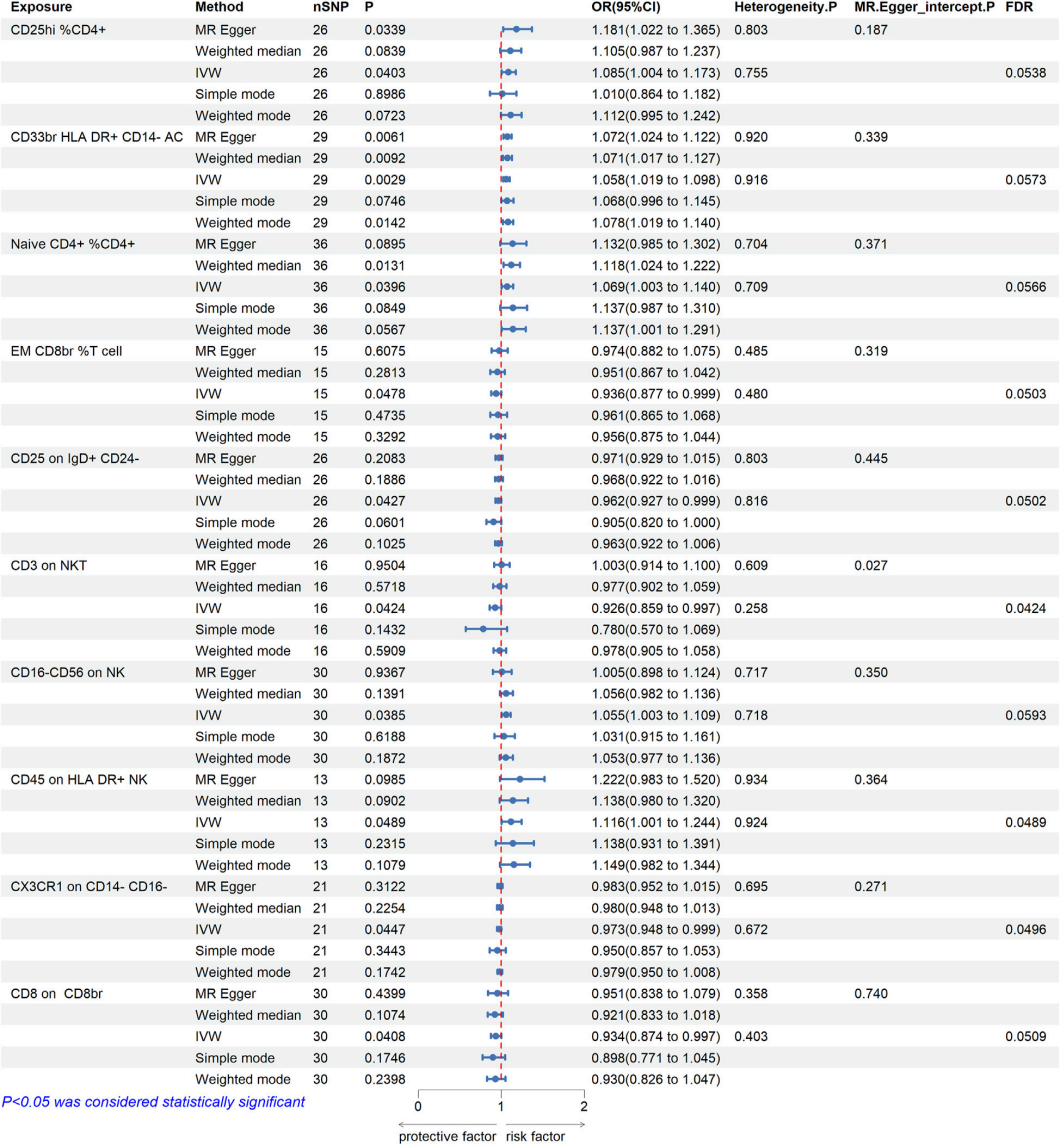
Mendelian randomization estimates of the association between immune cell phenotypes and risk of cerebral aneurysm. A total of 10 causal immune cell phenotypes were included (FDR < 0.06), three of which were significantly associated with CAs (CD3 on NKT, CD45 on HLA DR+ NK, and CX3CR1 on CD14- CD16-). OR, odds ratio; CI, confidence interval. IVW, inverse variance weighted.

In order to explore the causal effect of immunophenotypes on CAs, two-sample MR analysis was used, and IVW method was used as the main analysis method. The CD8br NKT % lymphocyte was not included because of the substantial variability observed in both the Cochran Q test and MR-PRESSO global test. After multiple correction using FDR method, 10 immune cell phenotypes had causal effects on the risk of CAs (FDR < 0.06), including three immune cell phenotypes with significant association (FDR < 0.05). Figure 1 illustrates that the IVW approach revealed a correlation between 10 immune cell phenotypes and the susceptibility to CAs. For FSC-A on lymphocyte, the Cochran Q test did detect significant heterogeneity (Supplementary Table S23), although there was some heterogeneity, it did not affect the result of the IVW method, and the result was robust. However, for other immune cell phenotypes, the Cochran Q test did not detect any significant heterogeneity, thus a fixed-effect model was employed to estimate the MR Effect size (Burgess et al., 2019). A protective effect was suggested by the negative correlation of 2 immune cell phenotypes (CD3 on NKT and CX3CR1 on CD14- CD16-). Using the IVW method, the odds ratio (OR) of CD3 on NKT for brain aneurysm risk was estimated to be 0.926 (95%CI = 0.859 to 0.997, *p* = 0.0424, FDR = 0.0424). However, other methods such as MR Egger (OR = 1.003, 95%CI = 0.914–1.100, *p* = 0.9504), Weighted median (OR = 0.977,95%CI = 0.902–1.059, *p* = 0.5718), Simple mode (OR = 0.780, 95%CI = 0.570–1.069, *p* = 0.1432), and Weighted mode (OR = 0.978, 95%CI = 0.905–1.058, *p* = 0.5909) did not yield similar results. The disease risk was positively correlated with the phenotype of the remaining immune cell (CD45 on HLA DR+ NK). Using the IVW method, the OR value of CD45 on HLA DR+ NK for the risk of CAs was estimated to be 1.116 (95%CI = 1.001–1.244, *p* = 0.0489, FDR = 0.0489). MR-Egger intercept showed no significant statistical results of horizontal pleiotropy (except for CD3 on NKT (*p* = 0.027) and Unsw mem % B cell (*p* = 0.024), all *p* > 0.05). Despite their pleiotropy, they were not estimated to be significant after outlier-corrected. Therefore, our results are robust. No indication of genetic pleiotropy bias was found in the sensitivity analysis. Supplementary Table S23 contains the findings from the heterogeneity test, pleiotropy test and MR-PRESSO global test.

### Causal effect of CAs risk on immune cell phenotypes

**FIGURE 2:**
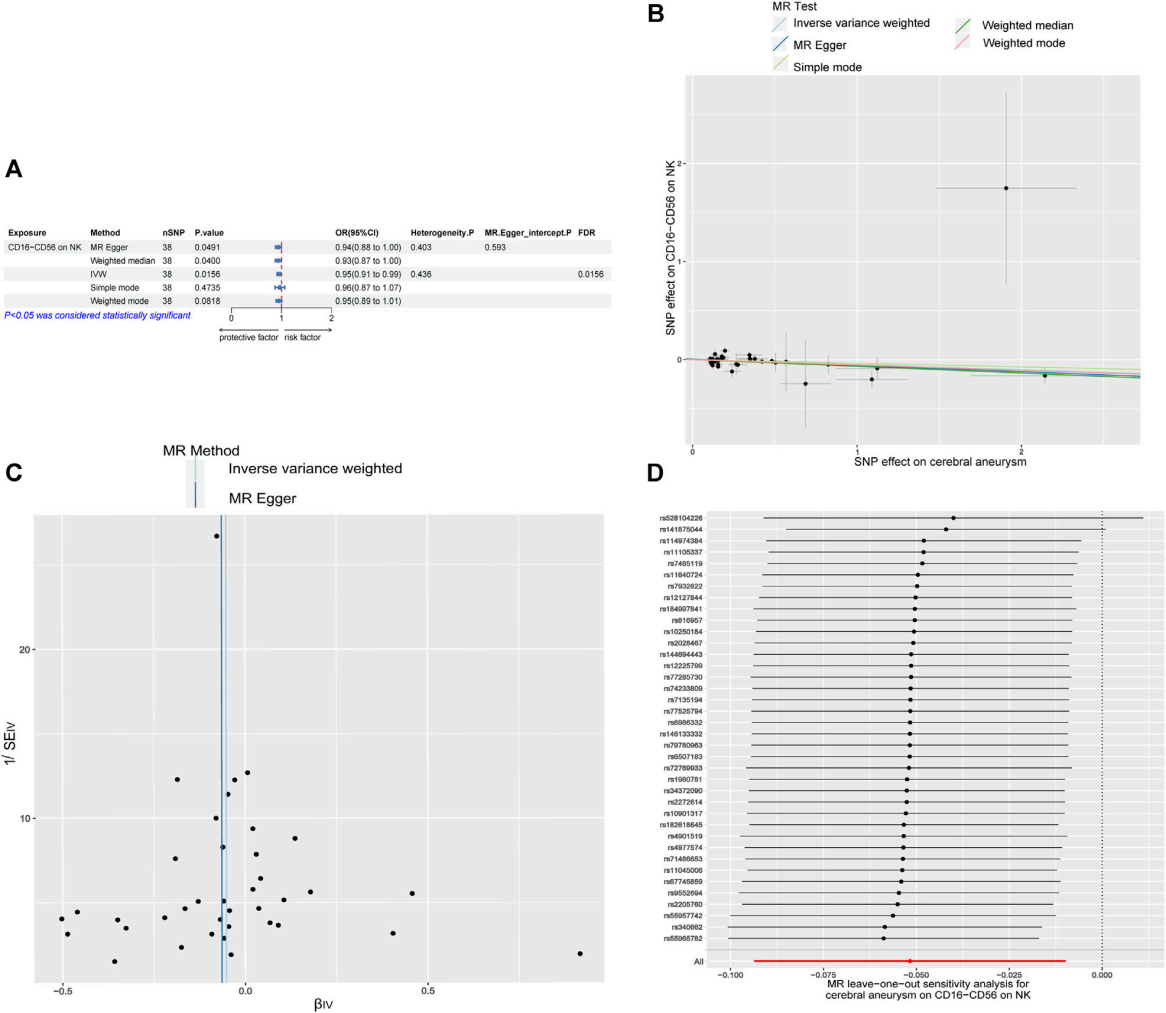
Causal relationship between immune cell phenotypes and CAs in reverse MR **(A)** Mendelian randomization estimates of the association between CD16-CD56 on NK cell and CAs. **(B)** Scatter plot of the causal effect of CD16-CD56 on NK cell on CAs risk. **(C)** Funnel plot of the causal effect of CD16-CD56 on NK cell on CAs risk. **(D)** Sensitivity analysis of the causal effect of CD16^−^CD56 on NK cell on CAs risk. OR, odds ratio; CI, confidence interval; IVW, inverse variance weighted.

Following that, a two-way MR investigation was carried out to explore the possible cause-and-effect connection between the risk of CAs and the characteristics of immune cells. This study utilized the 22 immunocell phenotypic SNPs previously associated with CAs. According to the IVW method, the results showed that there is an inverse relationship between the occurrence of CAs and the presence of CD16-CD56 on natural killer (NK) cells, with an odds ratio (OR) of 0.950 and a 95% confidence interval (CI) of 0.911–0.990, indicating statistical significance (*p* = 0.0156, FDR = 0.0156). The findings from Figure 2A were in line with the MR Egger and weighted median methods, whereas the simple mode and weighted mode did not provide evidence for this outcome. The confirmation of the results’ stability was further supported by the utilization of scatter plot and funnel plot (Figures 2B, C). The sensitivity analysis (Figure 2D) did not reveal any indication of bias towards genetic pleiotropy.

## Discussion

In this study, Mendelian randomization was used to examine the causal association between 731 immune cell characteristics and CAs. Utilizing publicly available genetic data, we identified four immune traits (MFI, RC, AC, and MP) that exhibited causal associations with 10 immune cell phenotypes in relation to CAs (FDR < 0.06), and three immune cell phenotypes had significant causal effects on CAs (FDR < 0.05). Conversely, only one immune cell phenotype demonstrated a significant causal association with CAs in reverse studies (FDR < 0.05).

Natural killer cells, also known as NK cells, are a varied group of lymphocytes that have the ability to bridge the gap between innate and adaptive immune responses (Alter and Altfeld, 2006). The absence of T cell or B cell receptors characterizes these cells, which can be divided into two main subgroups depending on the varying levels of CD16 and CD56 (Kucuksezer et al., 2021). NK cells display innate cytotoxicity, antibody-mediated cytotoxicity (ADCC), and have the ability to release multiple cytokines that resemble well-known CD4 helper T cell (Th) subsets, such as Th1, Th2, and Th17 (Chin et al., 2022). Inhibitory characteristics are possessedby NK cells that generate interleukin-10, potentially contrib an inverse relationship uting to immunomodulatory responses (Tahrali et al., 2018). Moreover, it has been noted that individuals with CAs exhibit notably reduced levels of CD8+T cells in their peripheral blood in comparison to those in the normal control group (Fan et al., 2020). Several research studies have presented evidence suggesting that inflammation plays a vital part in the development of CAs. The occurrence and development of CAs are regulated by negative regulatory molecules, like Tim-3, which modulate TNF-α in CD3^+^, CD4^+^, and CD8+T cells (Zhang et al., 2015). We found that CD3 on NKT cell was causally associated with a reduced risk of CAs. In a previous clinical study, increased percentages of CD3^+^ and NKT cell was found in patients with good prognosis after aSAH (Zhou et al., 2017). Our results also revealed that CAs increased CD16^−^CD56 on NK cell levels, and then there was a positive genetic causal link between CD45 on HLA DR+ NK cell and CAs. Previous studies have shown that individuals with CAs demonstrate impaired immune function, irregularities in CD4+T cell clusters, elevated levels of B cells, and increased expression of HLA-DR in monocytes, decreased levels of CD8+T cell, DNT cell, and DPT cell, along with heightened expression of TLR4, p-STAT3, and the depletion marker PD1 in NK cells (Ge et al., 2022). CX3CR1 on CD14- CD16- derived from monocytes has been shown to be associated with the risk of developing CAs (Mohme et al., 2020). This is consistent with our conclusions.

B lymphocytes have been recognized as important contributors to the development of persistent inflammation (Ma et al., 2021). According to research, the cytokine IL-6 plays a role in both the inflammation and development of B cells (Dmitrieva et al., 2016). Furthermore, there has been anoted elevation in IL-6 levels within the tissues of individuals afflicted with abdominal aortic aneurysms and intracranial aneurysms (Wang et al., 2018). Furthermore, the detection of T and B lymphocytes, dendritic cells (DC), and pericytes inside the blood vessels of CAs at the individual cell level in mice implies the participation of antigen-triggered stimulation and adaptive immune reactions of inexperienced T cells in the advancement of elastase-induced CAs. The discovery of these cells has revealed their crucial involvement in the rupture of CAs. Our results also cannot confirm that B and T lymphocytes increased the risk of CAs, and more studies are needed to investigate.

Additionally, it is important to note that neutrophils have a vital function in the context of CAs (Kushamae et al., 2020). Multiple research studies have shown that machine learning algorithms can be used to forecast the existence of CAs in blood samples from CAs patients by identifying the levels of neutrophil RNA expression (Tutino et al., 2018). Similarly, multiple research studies offer proof that supports the connection between the ratio of neutrophils to lymphocytes (NLR) and the outlook for patients with CAs, indicating the potential importance of NLR in predicting outcomes for this group (Cho et al., 2020; Zhang et al., 2021; Zhang et al., 2023). However, our results cannot show a causal effect between neutrophils and CAs, and more evidence is still needed to explore their relationship.

Our research has certain limitations. Initially, the individuals involved in this research were limited to the European population, and there was no personal information including age, sex, and consequently constraining the applicability of our discoveries to the wider population. Additional investigation is required to confirm and enhance the identified correlation. Secondly, the threshold filtering of the independent variable (IV) yielded a significance level of *p* < 1.0 × 10^−5^, which is relatively low. Third, we did not use multivariate MR Method and added additional data to further validate our findings, so additional evidence is necessary to further investigate the association between immune cell phenotypes and CAs. Moreover, it is crucial to recognize the intricacy of the cellular immune system, where the phenotype of immune cells functions as an indicator or guide. Further research should focus on examining particular subsets of immune cells and their roles in the formation of CAs.

## Conclusion

To summarize, our research provides preliminary proof that there is a cause-and-effect relationship between immune cell characteristics and CAs by utilizing MR techniques. The potential of this finding is to bring forth fresh viewpoints and strategies for the prevention and control of CAs. However, further inquiries are necessary to confirm and clarify the complex mechanisms that contribute to the participation of immune cells inthe development of CAs.

## Data Availability

The original contributions presented in the study are included in the article/Supplementary Material, further inquiries can be directed to the corresponding author.
